# Knockdown of SEMA7A alleviates MPP^+^‐induced apoptosis and inflammation in BV2 microglia via PPAR‐γ activation and MAPK inactivation

**DOI:** 10.1002/iid3.756

**Published:** 2023-01-13

**Authors:** Weinan Qi, Dan Zeng, Xiaoshuan Xiong, Qun Hu

**Affiliations:** ^1^ Department of Neurology Yantian District People's Hospital Shenzhen China; ^2^ Department of Radiology Yantian District People's Hospital Shenzhen China; ^3^ Department of Cardiology Yantian District People's Hospital Shenzhen China; ^4^ Department of Anesthesiology Yichun People's Hospital Yichun China

**Keywords:** apoptosis, inflammation, MAPK, microglia, PPAR‐γ, SEMA7A

## Abstract

**Introduction:**

The inflammation mediated by microglial cells plays an important role in the process of neurodegenerative diseases. Recent evidence indicates that semaphorin 7A (SEMA7A) is implicated in various neurodegenerative diseases, but whether it plays a role in Parkinson's disease (PD) remains unclear.

**Methods:**

In this study, 1.0 mmol/L 1‐methyl‐4‐phenylpyridinium (MPP^+^)‐stimulated mouse microglia (BV2) cells were used as an in vitro model of PD. The expression of SEMA7A was detected by quantitative polymerase chain reaction. Cell Counting Kit‐8 and apoptosis kits were used to analyze the viability and apoptosis of BV‐2 cells. The content of IL‐6, IL‐β, and tumor necrosis factor‐α was determined by ELISA (enzyme‐linked immunosorbent assay) kit. Western blot was used to detect the protein expression level of the inducible NO synthase and cyclooxygenase‐2.

**Results:**

Our findings indicated that SEMA7A expression in BV2 cells was upregulated after MPP^+^ stimulation. Knockdown of SEMA7A promoted cell viability while it inhibited apoptosis and the expression of proinflammatory enzymes and proinflammatory cytokines. Silencing SEMA7A‐induced peroxisome proliferator‐activated receptor‐gamma (PPAR‐γ) activation and mitogen‐activated protein kinase (MAPK) signaling pathway inactivation. Furthermore, a PPAR‐γ inhibitor and an MAPK activator promoted the effect of MPP^+^ on cell viability, apoptosis, and inflammation of BV2 cells; what is more, the PPAR‐γ inhibitor and MAPK activator blocked the inhibitory effect of SEMA7A downregulation on MPP^+^‐induced injury.

**Conclusion:**

In general, knockdown of SEMA7A inhibits MPP^+^‐induced BV2 cell apoptosis and inflammation via PPAR‐γ activation and MAPK inactivation, which may provide a new therapy target for PD.

## Graphical abstract

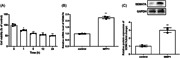

## 1 INTRODUCTION

Neuroinflammation is closely related to various neurodegenerative diseases, including Parkinson's disease (PD).^1^ Increasing evidence shows that excessively activated microglial cells produce large amounts of proinflammatory cytokines, such as tumor necrosis factor‐α (TNF‐α), interleukins (such as interleukin 1β [IL‐1β] and interleukin‐6 [IL‐6]), and inducible NO synthase (iNOS), which cause the nervous system damage and lead to neuroinflammation.^2^, ^3^, ^4^ It has been reported that microglia can be activated by 1‐methyl‐4‐phenylpyridinium (MPP^+^), an active metabolite of the neurotoxic substance 1‐methyl‐4‐phenyl‐1,2,3,6‐tetrahydropyridine (MPTP) that is frequently used in establishment of neuroinflammation models in vitro,^5^, ^6^, ^7^ but the molecular mechanism involved in MPP^+^‐activated microglia is remain largely unknown.

Semaphorins are axon‐directed molecular proteins, a family of secreted or transmembrane proteins that play a variety of important functions in the peripheral nervous system and central nervous system.^8^, ^9^, ^10^ The researches have illustrated that semaphorin 7A (SEMA7A) not only promotes the inflammatory response by inducing proinflammatory cytokines (such as TNF‐α and IL‐6) but also acts as an effective autocrine stimulator of monocytes, stimulating inflammatory cytokines production and superoxide release.^11^ In addition, previous studies have indicated that SEMA7A initiates an inflammatory response by inducing phosphorylation of ERK 1/2 in human monocyte cell lines.^12^ This evidence implies that SEMA7A plays a crucial role in the inflammatory response. What is more, SEMA7A is reported to be involved in central nervous system inflammation in experimental autoimmune encephalomyelitis and multiple sclerosis, and SEMA7A can also activate mitogen‐activated protein kinase (MAPK) signaling cascade pathways to affect the formation of neuronal networks in neuronal cells.^13^, ^14^ These characteristics of SEMA7A indicate that it plays a role in regulating inflammation of the nervous system; however, the role of SEMA7A in microglia remains unclear.

In this study, we aimed to explore the function and underlying mechanism of SEMA7A in an MPP^+^‐induced cell model of PD. The present study demonstrates that SEMA7A might be able to influence apoptosis and inflammation of MPP^+^‐activated microglia injury through the MAPK signaling pathway though MAPK inactivation and peroxisome proliferator‐activated receptor‐gamma (PPAR‐γ) activation.

## 2 MATERIALS AND METHODS

### 2.1 Cell culture

The mouse microglia cell line BV2 was purchased from the Institute of Basic Medical Sciences of the China Science Academy. BV2 cells were cultured in DMEM medium (Invitrogen) supplemented with 10% fetal bovine serum (Thermo Fisher Scientific), 100 U/ml penicillin (MilliporeSigma), and 100 μg/ml streptomycin (Invitrogen). The cells were incubated in a humidified incubator, at 37°C, in 5% CO_2_ and 95% air.

### 2.2 Cell transfection

The small interfering RNA against SEMA7A (si‐SEMA7A) and its negative controls (si‐NC) were all synthesized by GenePharma Co., Ltd. Then, BV2 cells were harvested for the subsequent experiments after transfection by Lipofectamine 3000 reagent (Invitrogen) for 48 h, according to the manufacturer's protocol.

### 2.3 Cell viability assay

The treated or nontreated BV2 cells were seeded into 96‐well plates at a density of 1 × 10^4^/per well. After 24 h of incubation, 10 µl of Cell Counting Kit‐8 (CCK‐8; Dojindo Laboratories) solution was pipetted in each well. Then, the cells were cultured for another 2 h, and the absorbance of each well's contents was measured by a SpectraMax M5 fluorimeter at 450 nm.

### 2.4 Detection of apoptosis

The treated or nontreated BV2 cells were seeded into 96‐well plates at a density of 1 × 10^4^/per well. After 24 h of incubation, experiments were performed in compliance with the instructions in the cell death enzyme‐linked immunosorbent assay (ELISA) (Roche Mannheim Biochemicals).

### 2.5 5‐bromo‐2*′*‐deoxyuridine (BrdU) cell proliferation assay

Cell proliferation was detected using the BrdU method. Briefly, transfected cells were cultured in 12‐well plates with appropriate density. Twelve hours later, experiments were performed in compliance with the instructions in the BrdU kit (Invitrogen). Images were acquired using fluorescence microscopy and the relative proportion of BrdU‐positive cells was calculated.

### 2.6 Quantitative real‐time polymerase chain reaction (qRT‐PCR)

All RNA in BV2 cells was isolated using TRIzol reagent (Takara). Isolated RNA (1 μg) was synthesized to cDNA using a Bestar™ qPCR RT kit (DBI). RT‐qPCR analyses were performed with Bestar™ qPCR MasterMix (DBI) on an Agilent Stratagene Mx3000P fluorescence quantitative PCR instrument. Gene expression was analyzed by the $2^{‐∆∆C_{t}}$ method. The primer sequences used were:

iNOS (forward): 5′‑CTGATGTTGCCATTGTTGGTG‑3′,

iNOS (reverse): 5′‑CTTTGACGCTCGGAACTGTAG‑3′;

COX‐2 (forward): 5′‑GGAGAGACTATCAAGATAGT‑3′,

COX‐2 (reverse): 5′‑ATGGTCAGTAGACTTTTACA‑3′;

TNF‐α (forward): 5′‐TTCGAGTGACAAGCCTGTAGC‐3′,

TNF‐α (reverse): 5′‐AGATTGACCTCAGCGCTGAGT‐3′;

IL‐1β (forward): 5′‐AATCTCACAGCACATCAA‐3′,

IL‐1β (reverse): 5′‐AGCCCATACTTTAGGAAGACA‐3′;

IL‐6 (forward): 5′‐GAGGATACCACTCCCAACAGACC‐3′,

IL‐6 (reverse): 5′‐AAGTGCATCATCGTTGTTCATACA‐3′;

GAPDH (forward): 5′‐CCGCATCTTCTTGTGCAGTG‐3′,

GAPDH (reverse): 5′‐ATGAAGGGGTCGTTGATGGC‐3′.

### 2.7 ELISA

The transfected and untransfected BV2 cells were induced by 1 μg/ml MPP^+^ for 12 h. The levels of TNF‐α, IL‐1β, and IL‐6 were detected by ELISA kit (R&D Systems) in cells supernatant according to the protocols of the manufacturer's protocols. The absorbance of all samples was measured using a microplate reader (Multiskan Spectrum; Thermo Fisher Scientific). Cytokine levels were standardized as protein concentrations.

### 2.8 Western blot analysis

After treatment, BV2 cells were collected, and the total protein was extracted after lysing with RIPA lysis buffer (Beyotime Biotechnology, Inc.). Total tissue or cellular proteins were separated by sodium dodecyl sulfate polyacrylamide gel electrophoresis (SDS‐PAGE) and proteins were transferred to PVDF membranes (Millipore). Then, membranes were incubated with primary and secondary antibodies followed by visualization of the blots with ECL chemiluminescent solution (Advasta). The primary antibody was obtained from Abcam; the dilution ratio was 1:1000.

### 2.9 Statistical analysis

All data collected from three replicate experiments are presented as the mean ± standard deviation. Statistical differences between two and more than two groups were analyzed by Student's *t*‐test and one‐way analysis of variance, respectively. *p* < .05 represents a statistically significant difference.

## 3 RESULTS

### 3.1 SEMA7A expression was increased by MPP^+^ treatment in BV2 cells

**Figure 1:**
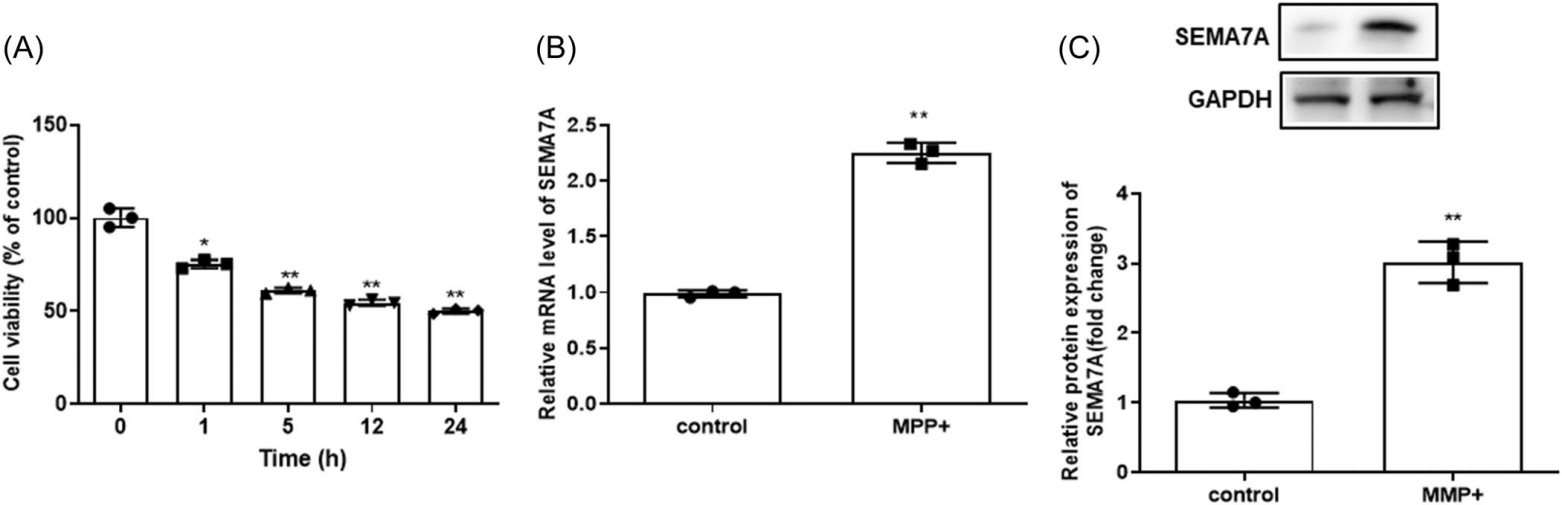
SEMA7A expression was increased by MPP^+^ treatment in BV2 cells. (A) CCK‐8 kit was used to analyze the cell viability of BV2 cells after treated with 1 μg/ml MPP^+^ for different durations (0, 1, 5, 12, or 24 h). *p < .05 versus 0 h, **p < .01 versus 0 h. (B) The qRT‐PCR analysis of SEMA7A expression in BV2 cells after treatment with MPP^+^ for 12 h, with untreated BV2 cells as control. *p < .05 versus control. (C) The Western blot analysis of SEMA7A expression in BV2 cells after treatment with MPP^+^. *p < .05 versus control. *p < .05 versus control. CCK‐8, Cell Counting Kit‐8; MPP^+^, 1‐methyl‐4‐phenylpyridinium; qRT‐PCR, quantitative real‐time polymerase chain reaction; SEMA7A, semaphorin 7A.

We initially determined the expression level of SEMA7A in an MPP^+^‐treated microglial cell line, BV2. Figure 1A indicates that cell viability was reduced by 1.0 mmol/L. MPP^+^ treatment also affected cell viability in a time‐dependent manner (*p* < .05). Based on the above results, treatment for 24 h was chosen for the subsequent experiments. Then, the results of qRT‐PCR and Western blot showed that SEMA7A expression of BV2 cells was significantly increased by MPP^+^ treatment (*p* < .05, Figure 1B,C).

### 3.2 Knockdown of SEMA7A attenuated MPP^+^‐induced apoptosis in BV2 cells

**Figure 2:**
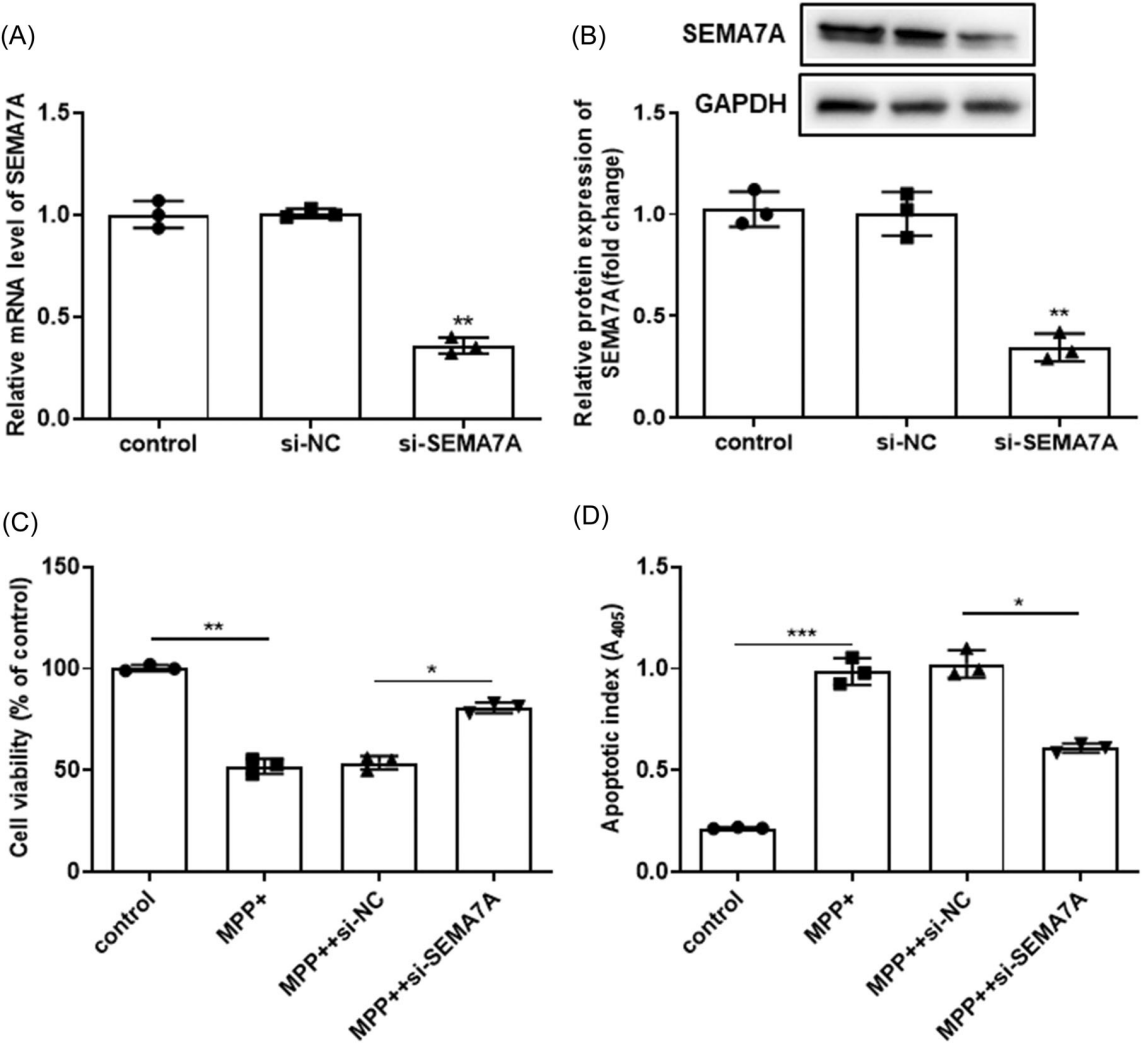
Knockdown of SEMA7A attenuated MPP^+^‐induced apoptosis in BV2 cells. (A, B) The mRNA and protein expression of SEMA7A of BV2 cells after the small interfering RNA against SEMA7A (si‐SEMA7A) and its control (si‐NC) transfection. (C) The BV2 cells were transfected with si‐SEMA7A and si‐NC before MPP^+^ treatment. The viability of BV2 cells was assessed by CCK‐8 assay, with untreated BV2 cells as control. *p < .05. (D) The BV2 cells were transfected with si‐SEMA7A and si‐NC before MPP^+^ treatment. The apoptotic rate of BV2 cells was detected by a cell death enzyme‐linked immunosorbent assay, with untreated BV2 cells as control. *p < .05. CCK‐8, Cell Counting Kit‐8; GAPDH, glyceraldehyde 3‐phosphate dehydrogenase; MPP^+^, 1‐methyl‐4‐phenylpyridinium; mRNA, messenger RNA; SEMA7A, semaphorin 7A.

We next explored the effect of aberrant SEMA7A expression on BV2 cell viability and apoptosis after MPP^+^ stimulation. As shown in Figure 2A,B, the si‐SEMA7A significantly reduced the messenger RNA (mRNA) and protein expression of SEMA7A in BV2 cells. Further data demonstrated that si‐SEMA7A enhanced cell viability while it inhibited apoptosis in MPP^+^‐treated BV2 cells (*p* < .05, Figure 2C,D). Therefore, to explore whether the knockdown of SEMA7A in microglia is conducive to the survival of DA neurons, we will co‐culture microglia and neurons to explore the impact of SEMA7A knockout on neurons. It was found that knockdown of SEMA7A in microglia decreased the cell viability of neurons and promoted the apoptosis of neurons (Supporting Information: Figure S1). Our results above preliminarily indicate that the knockdown of SEMA7A in microglia is not conducive to the survival of neurons.

### 3.3 Knockdown of SEMA7A attenuated MPP^+^‐induced inflammatory injury in BV2 cells

**Figure 3:**
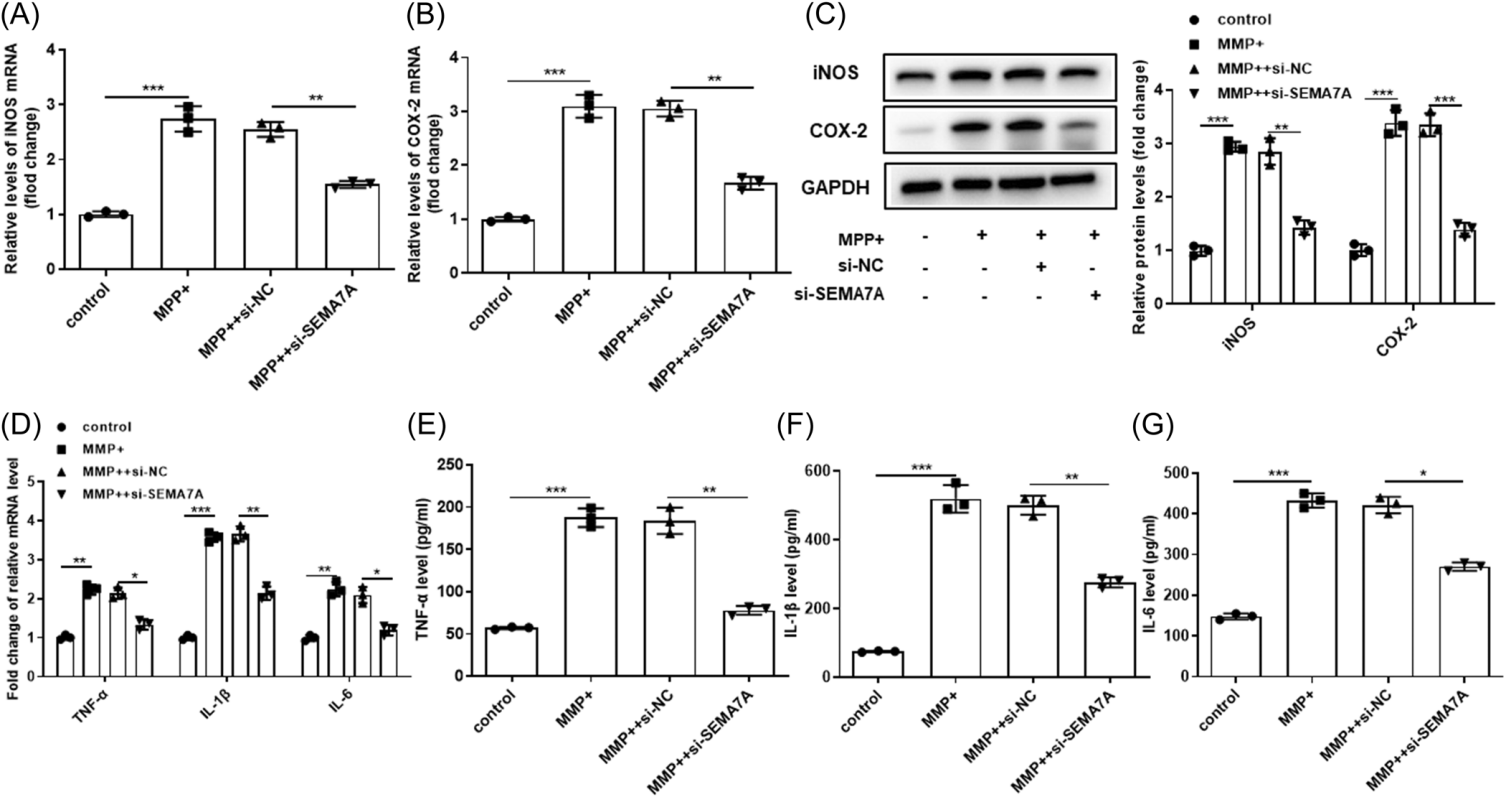
Knockdown of SEMA7A attenuated MPP^+^‐induced inflammatory injury in BV2 cells. The BV2 cells were transfected with si‐SEMA7A and si‐NC before MPP^+^ treatment, and nontreated BV2 cells were used as control. (A, B) The mRNA expression of proinflammatory enzymes iNOS and COX‐2 in BV2 cells were detected by qRT‐PCR. *p < .05. (C) The Western blot analysis of iNOS and COX‐2 protein expression. *p < .05. (D) The mRNA expression of TNF‐α, IL‐1β, and IL‐6 mRNA in BV2 cells were detected by qRT‐PCR. (E–G) The protein concentration of TNF‐α, IL‐1β, and IL‐6 mRNA in BV2 cells were detected by enzyme‐linked immunosorbent assay. GAPDH was used as control. *p < .05; **p < .01; ***p < .001. COX‐2, cyclooxygenase‐2; GAPDH, glyceraldehyde 3‐phosphate dehydrogenase; IL‐1β, interleukin 1β; IL‐6, interleukin‐6; iNOS, inducible NO synthase; MPP^+^, 1‐methyl‐4‐phenylpyridinium; mRNA, messenger RNA; TNF‐α, tumor necrosis factor α; qRT‐PCR, quantitative real‐time polymerase chain reaction; SEMA7A, semaphorin 7A.

The present study further investigated the involvement of SEMA7A in inflammatory regulation. The qRT‐PCR and Western blot results showed that si‐SEMA7A attenuated MPP^+^‐increased iNOS and cyclooxygenase‐2 (COX‐2) expression levels in BV2 cells (*p* < .05, Figure 3A,C). The results shown in Figure 3D,G illustrate that MPP^+^ stimulation conspicuously elevated the mRNA expression levels and concentrations of TNF‐α, IL‐1β, and IL‐6 in BV2 cells, while si‐SEMA7A inhibited this promotion in BV2 cells (*p* < .05).

### 3.4 Knockdown of SEMA7A decreased MPP^+^‐induced MAPK activation and PPAR‐γ inactivation in BV2 cells

**Figure 4:**
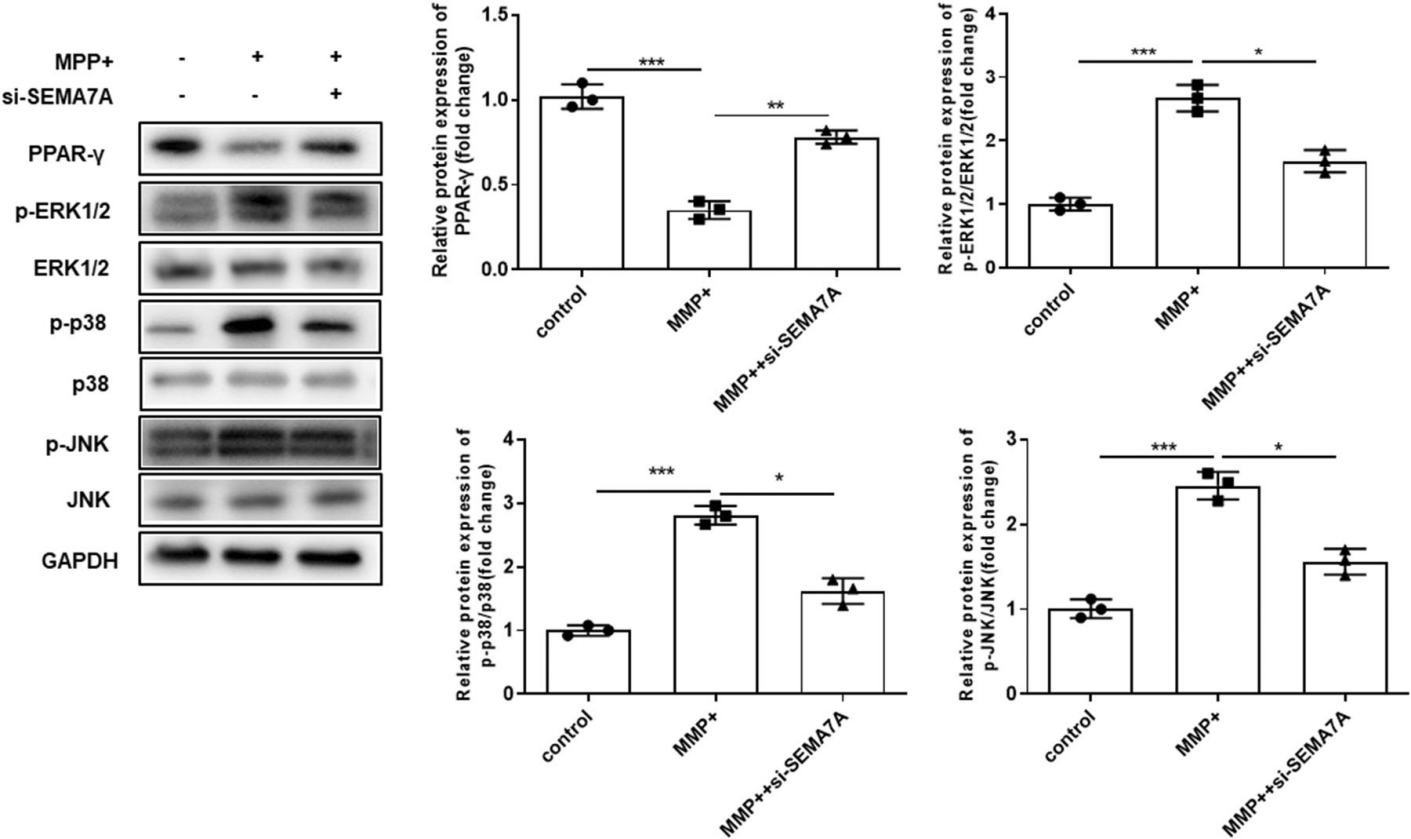
Knockdown of SEMA7A decreased MPP^+^‐induced MAPK activation and PPAR‐γ inactivation in BV2 cells. The BV2 cells were transfected with si‐SEMA7A and si‐NC before MPP^+^ treatment, and nontreated BV2 cells were used as control. The protein expression of PPAR‐γ, and MAPK signaling pathway factors, including p‐ERK1/2, ERK1/2, p‐p38, p38, p‐JNK, JNK, were analyzed by Western blot, respectively. GAPDH was used as control. *p < .05; **p < .01; ***p < .001. GAPDH, glyceraldehyde 3‐phosphate dehydrogenase; MAPK, mitogen‐activated protein kinase; MPP^+^, 1‐methyl‐4‐phenylpyridinium; PPAR‐γ, peroxisome proliferator‐activated receptor‐gamma; SEMA7A, semaphorin 7A.

It has been proved that peroxisome PPAR‐γ activation contributes to the suppression of microglial inflammation.^15^, ^16^ Moreover, several semaphorin members have been proved to interact with PPAR‐γ in diverse cells.^17^, ^18^, ^19^ Our work indicated that si‐SEMA7A reduced the improvement of MPP^+^ stimulation on PPAR‐γ expression in BV2 cells (*p* < .05, Figure 4). What is more, SEMA7A has been proved to activate MAPK signaling cascade pathways to participate in the formation of neuronal networks.^13^, ^14^ Thus, we further investigated the relationship between SEMA7A and the MAPK signaling pathway in MPP^+^‐induced BV2 cells. Our data demonstrate that si‐SEMA7A suppressed the expression of MPP^+^‐activated MAPK signaling pathway factors, including p‐ERK1/2, ERK1/2, p‐p38, p38, p‐JNK, and JNK, in BV2 cells (*p* < .05, Figure 4).

### 3.5 MAPK activation and PPAR‐γ inactivation diminished the effect of SEMA7A knockdown on apoptosis in MPP^+^‐treated BV2 cells

**Figure 5:**
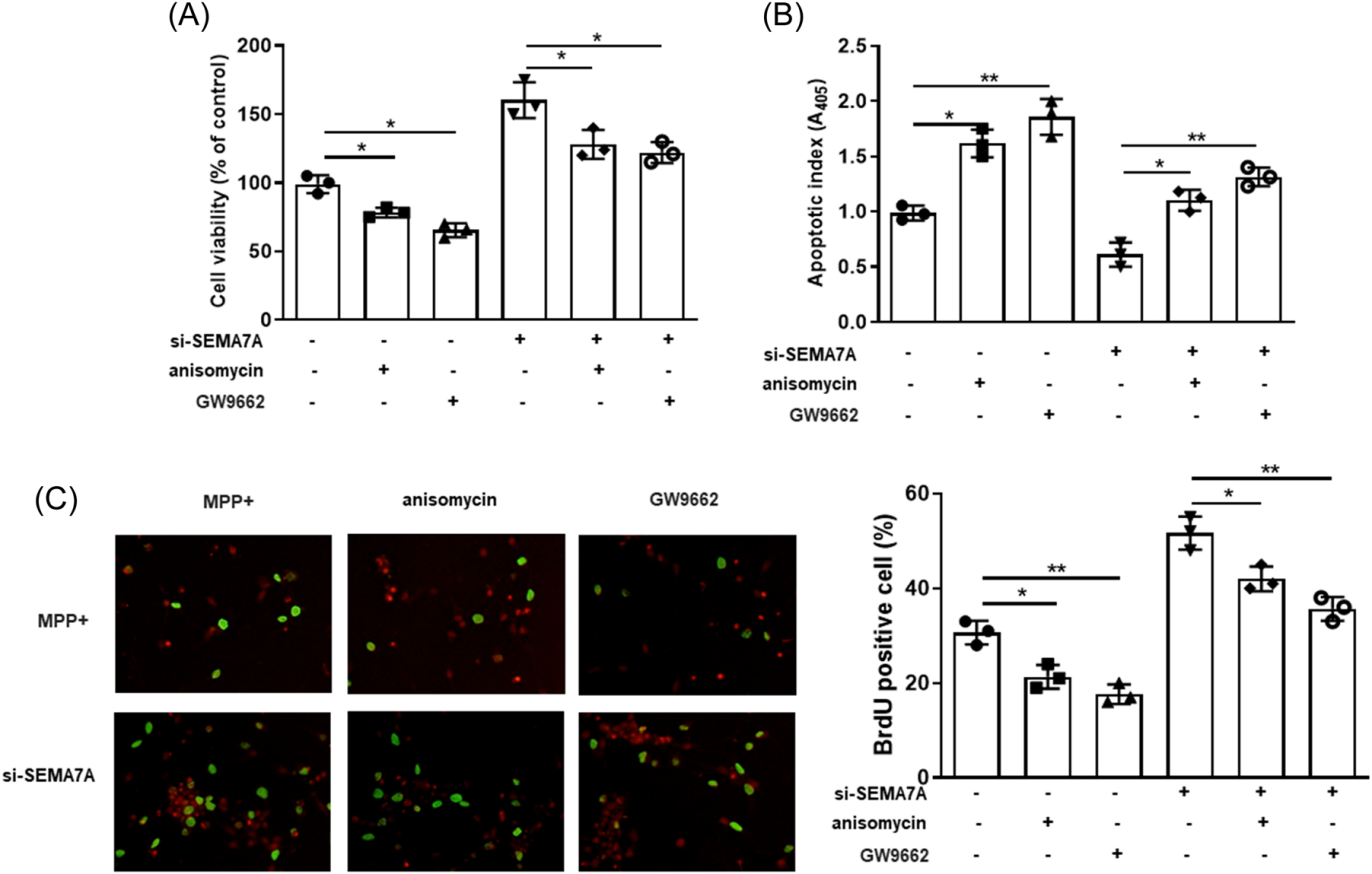
MAPK activation and PPAR‐γ inactivation diminished the effect of knockdown of SEMA7A on apoptosis in MPP^+^‐induced BV2 cells. BV2 cells were treated with si‐SEMA7A, anisomycin (an activator of the MAPK signaling pathway) or/and GW9662 (an antagonist of PPAR‐γ) before MPP^+^ stimulation. (A) The viability of BV2 cells was assessed by CCK‐8 assay. *p < .05. (B) The apoptotic rate of BV2 cells was detected by a cell death enzyme‐linked immunosorbent assay. *p < .05. (C) BrdU assay was applied to measure cell proliferation. The pictures (left panel) and numbers (right panel) of the BrdU positive cells were acquired under the fluorescence microscope (×200). *p < .05; **p < .01; ***p < .001. BrdU, 5‐bromo‐2*′*‐deoxyuridine; CCK‐8, Cell Counting Kit‐8; MAPK, mitogen‐activated protein kinase; MPP^+^, 1‐methyl‐4‐phenylpyridinium; PPAR‐γ, peroxisome proliferator‐activated receptor‐gamma; SEMA7A, semaphorin 7A.

The present study further explored the association between the MAPK signaling pathway, PPAR‐γ, and SEMA7A in BV2 cells. As illustrated in Figure 5, anisomycin (an activator of the MAPK signaling pathway) and GW9662 (an inhibitor of PPAR‐γ) markedly inhibited cell viability and cell proliferation while they promoted apoptosis in MPP^+^‐treated BV2 cells; what is more, anisomycin and GW9662 attenuated the effect of si‐SEMA7A on MPP^+^‐induced apoptosis in BV2 cells (*p* < .05). Hence, these findings reveal that downregulation of SEMA7A inhibited MPP^+^‐induced apoptosis of BV2 cells through PPAR‐γ activation and MAPK inactivation.

### 3.6 MAPK activation and PPAR‐γ inactivation diminished the effect of SEMA7A knockdown on inflammation in MPP^+^‐treated BV2 cells

**Figure 6:**
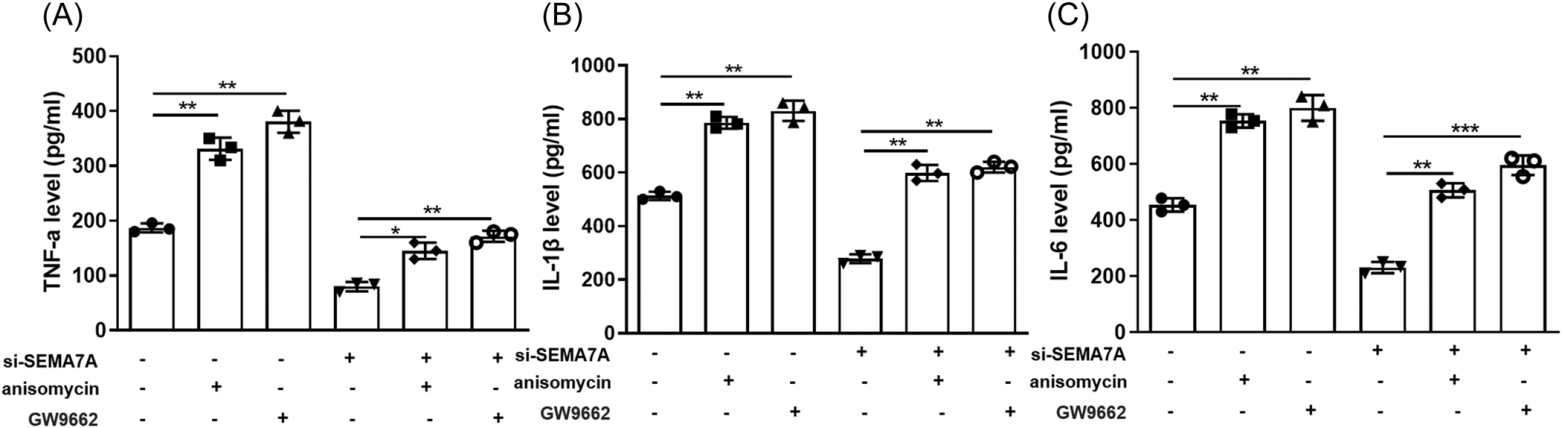
MAPK activation and PPAR‐γ inactivation diminished the effect of knockdown of SEMA7A on inflammation in MPP^+^‐induced BV2 cells. BV2 cells were treated with si‐SEMA7A, anisomycin (an activator of the MAPK signaling pathway) or/and GW9662 (an inhibitor of PPAR‐γ) before MPP^+^ stimulation. (A–C) The concentrations of TNF‐α, IL‐1β, and IL‐6 in BV2 cells were detected by enzyme‐linked immunosorbent assay. *p < .05; **p < .01; ***p < .001. IL‐1β, interleukin 1β; IL‐6, interleukin‐6; MAPK, mitogen‐activated protein kinase; PPAR‐γ, peroxisome proliferator‐activated receptor‐gamma; MPP^+^, 1‐methyl‐4‐phenylpyridinium; SEMA7A, semaphorin 7A; TNF‐α, tumor necrosis factor α.

The ELISA results also showed that anisomycin and GW9662 improved the concentration of MPP^+^‐induced TNF‐α, IL‐1β, and IL‐6 in BV2 cells; moreover, anisomycin and GW9662 further partly alleviated the effect of miR‐93‐5p mimics on expression of these cytokines in BV2 cells (*p* < .05, Figure 6A,C). Hence, these findings reveal that downregulation of SEMA7A inhibited MPP^+^‐induced inflammation of BV2 cells through PPAR‐γ activation and MAPK inactivation.

## 4 DISCUSSION

Elevated levels of proinflammatory mediators in activated microglia was proven to cause neurodegenerative disease, including PD.^20^ The microglia activated by MPP^+^ are frequently used as a cell model of neuroinflammation, and in this study, we found that MPP^+^ significantly suppressed cell viability while elevating the apoptosis rate and release of proinflammatory cytokines in mouse microglia BV2 cells.^7^, ^21^, ^22^ The present study shows that SEMA7A expression is decreased in MPP^+^‐treated^23^ BV2 microglia, and knockdown of SEMA7A suppresses MPP^+^‐induced apoptosis and inflammation in BV2 cells by activating PPAR‐γ and blocking the MAPK signaling pathway.

Current research indicates that SEMA7A plays an important role in the regulation of various inflammatory responses. Namavari et al.^24^ reported that SEMA7A contributes to nerve regeneration and inflammatory processes in the cornea. De Minicis et al.^25^ found that SEMA7A is highly expressed upon TGF‐β stimulation in hepatic stellate cells, and increased SEMA7A enhances expression of inflammatory factors. Previous studies have also demonstrated that deletion of SEMA7A has an anti‐inflammatory effect on seawater aspiration‐induced acute lung injury, collagen‐induced arthritis, myocardial tissue injury, and coxsackievirus B3‐induced viral myocarditis.^26^, ^27^ What is more, it has been reported that SEMA7A promotes growth and migration of squamous cell carcinoma (OTSCC) by regulating the TGF‐β‐induced EMT signaling pathway in OTSCC cells, which provides a new interconnection between SEMA7A and the TGF‐β‐induced EMT signaling pathway.^23^ In this study, we found that knockdown of SEMA7A suppresses MPP^+^‐induced apoptosis and inflammation in BV2 cells.

PPAR‐γ is a ligand‐activated transcription factor and member of the nuclear receptor superfamily, mainly expressed in monocytes/macrophages, adipocytes, and so forth.^16^ In the past decades, PPAR‐γ has been found to play a crucial role in various neurodegenerative diseases.^28^, ^29^, ^30^ Previous studies have found that PPAR‐γ expression is downregulated in lipopolysaccharide (LPS)‐stimulated BV2 cells, and activation of PPAR‐γ retards the LPS‐induced secretion of proinflammatory mediators and cell apoptosis.^15^, ^31^, ^32^ A recent study demonstrated that the PPAR‐γ antagonist GW9662 inhibits MPTP‐induced microglial activation in a mouse PD model.^33^ In addition, the PPAR‐γ agonist MDG548 attenuates the LPS‐stimulated microglial phenotype by suppressing inflammation, and further data indicate that MDG548 reverses the MPTP‐induced changes in cytokines in the microglia of mice.^34^ A growing body of evidence has proved that PPAR‐γ has neuroprotective effects on PD by inhibiting inflammation and apoptosis in microglia.^33^, ^35^, ^36^ Furthermore, it has been revealed that several semaphorin members play a role in regulating various inflammatory responses via interacting with PPAR‐γ.^17^, ^37^ In the present study, our findings suggest that silencing SEMA7A represses MPP^+^‐induced apoptosis and inflammation in microglia by activating PPAR‐γ.

MAPK, a type of protein kinase, is an important mediator that can be activated by extracellular stimulus signals, and it is implicated in diverse cellular processes, including development, differentiation, proliferation, and apoptosis.^38^ Data from clinical experiments demonstrate that activation of the MAPK signaling pathway is frequently observed in PD patients, suggesting it may play a role in PD progression.^39^ Further molecular mechanism studies indicate that the expression levels of apoptosis factors are downregulated by blocking MAPK signaling pathway activation in microglia.^40^ Moreover, the MAPK signaling pathway can participate in the production of proinflammatory factors in microglia that lead to chronic neuroinflammation.^41^, ^42^ A growing number of studies indicate that inhibition of MAPK signaling pathway activation diminishes apoptosis and inflammation in microglia.^43^, ^44^, ^45^ Our findings indicate that silencing SEMA7A represses MPP^+^‐induced apoptosis and inflammation in microglia by inactivating the MAPK signaling pathway.

## 5 CONCLUSION

This study demonstrates that SEMA7A is highly expressed in MPP^+^‐treated BV2 cells. Silencing of SEMA7A enhances MPP^+^‐decreased cell viability and alleviates MPP^+^‐increased apoptosis and the production of proinflammatory cytokines via MAPK signaling pathway inactivation and PPAR‐γ activation. Our research provides an understanding of microglia‐mediated neuroinflammation and a potential target for molecular therapy of neuroinflammation. Although this is a gratifying result, there are still some shortcomings in the current research. We only conducted related studies on BV2 cells. To more fully verify the role of SEMA7A, we also need to explore in vivo mouse experiments. Next, we will focus on whether the silencing of SEMA7A has the same effect in other related cells. Meanwhile, a mouse model was used to verify the role of SEMA7A in neuroinflammation progression.

## CONFLICT OF INTEREST

The authors declare no conflict of interest.

## Supporting information


**Fig.S1. Knockdown of SEMA7A reduced the cell viability and increase the MPP** ^+^ **‐induced apoptosis of neurons**.

## Data Availability

The data used to support the findings of this study are available from the corresponding author upon request.
