# Supplementary figures and images for: Knockdown of SEMA7A alleviates MPP+‐induced apoptosis and inflammation in BV2 microglia via PPAR‐γ activation and MAPK inactivation
Source: Immun Inflamm Dis. 2023 Jan 13;11(1):e756. doi: 10.1002/iid3.756 (PMC9837934; doi:10.1002/iid3.756)

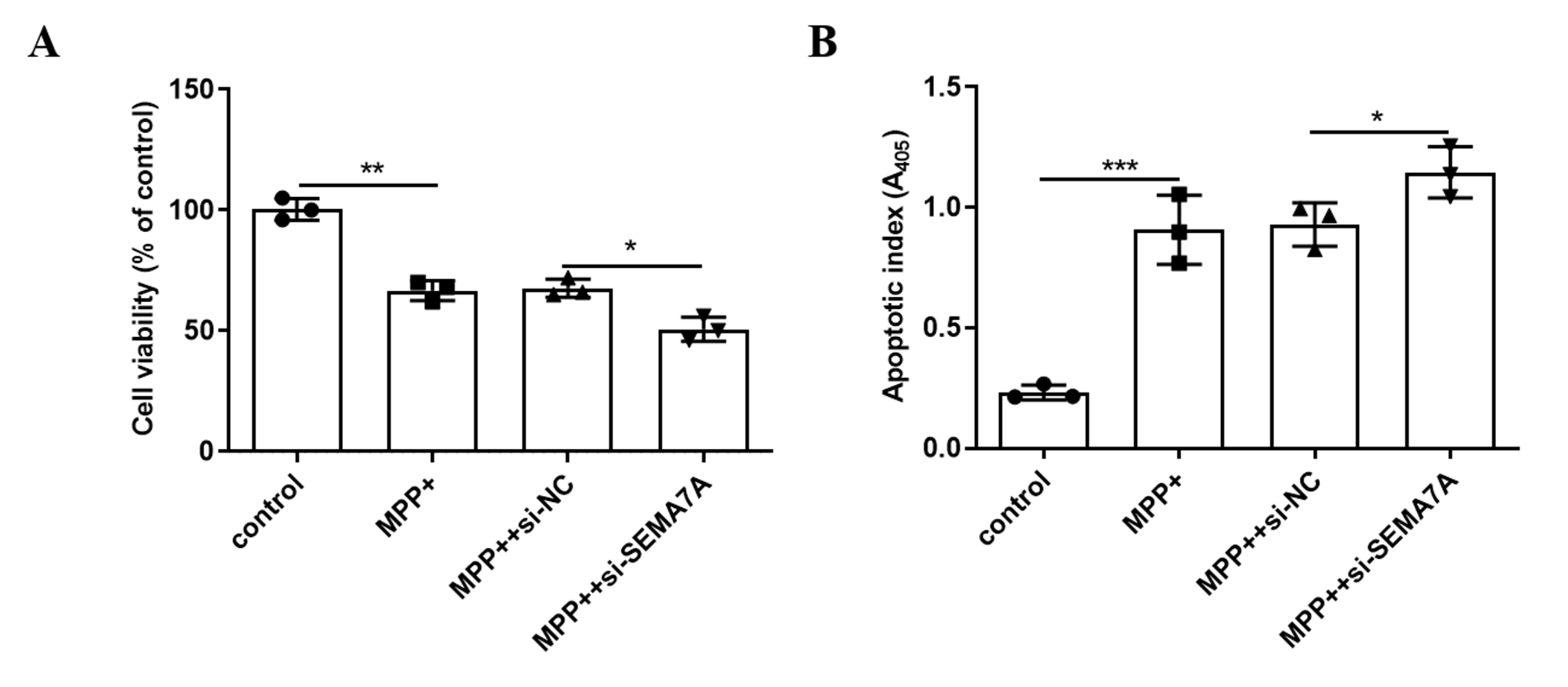

Supplement: Supplementary file 1 — Fig.S1. Knockdown of SEMA7A reduced the cell viability and increase the MPP + ‐induced apoptosis of neurons. [file IID3-11-e756-s001.tif]
